# Supplementary material for: Socio-demographic and neighbourhood factors influencing urban green space use and development at home: A population-based survey in Accra, Ghana
Source: PLoS One. 2023 Jun 23;18(6):e0286332. doi: 10.1371/journal.pone.0286332 (PMC10289371; doi:10.1371/journal.pone.0286332)
Supplement: S1 File — (DOCX) [file pone.0286332.s001.docx]

**Table S1.** Distribution of district by enumeration area (EA) and households (HH)

| **District** | **No. of HHs** | **Proportion** | **Sample HHs** | **Proportionate EAs Allocations** | **Adjusted EAs Allocation** | **Adjusted HHs** |
| --- | --- | --- | --- | --- | --- | --- |
| Ablekuma North | 54344 | 11.3 | 227 | 22.7 | 23 | 230 |
| Ablekuma West | 49031 | 10.2 | 205 | 20.5 | 20 | 200 |
| Ablekuma Central | 57446 | 12.0 | 240 | 24.0 | 24 | 240 |
| AMA | 98298 | 20.5 |  |  |  |  |
| Ablekuma South | 35821 | 7.5 | 150 | 15.0 | 15 | 150 |
| Ashiedu Keteke | 33572 | 7.0 | 140 | 14.0 | 14 | 140 |
| Okaikoi South | 28905 | 6.0 | 121 | 12.1 | 12 | 120 |
| Korle Klottey | 25558 | 5.3 | 107 | 10.7 | 11 | 110 |
| Ayawaso Central | 33149 | 6.9 | 138 | 13.8 | 14 | 140 |
| Ayawaso East | 16893 | 3.5 | 71 | 7.1 | 7 | 70 |
| Ayawaso North | 21150 | 4.4 | 88 | 8.8 | 9 | 90 |
| Ayawaso West | 22264 | 4.6 | 93 | 9.3 | 9 | 90 |
| La-Dadekotopon | 47515 | 9.9 | 198 | 19.8 | 20 | 200 |
| Okaikoi North | 53166 | 11.1 | 222 | 22.2 | 22 | 220 |
| **Total** | **478814** |  | **2000** | **200** |  | **2000** |

**Computation of Sampling Weights**

Due to the non-proportional allocation of the sample to the different regions and the possible differences in response rates, sampling weights will be required for any analysis using (survey name) data to ensure actual representation of the survey results at national and domain level. Since the (survey name) sample is a multi-stage stratified cluster sample, sampling weights will be calculated based on sampling probabilities separately for each sampling stage and for each cluster. We use the following notations

1. *P*_1_*_hi_*: first-stage sampling probability of the *i^th^* EA in stratum *h* from the sampling frame

*P*_2_*_hi_*: second -stage sampling probability within the *i^th^* EA (household selection)

Let$n_{h}$be the number of EAs selected in stratum *h*, *M_hi_* the measure of size (number of residential households) according to the sampling frame in the *i*^th^ EA, and $\sum M_{hi}$ the total measure of size (total number of residential households) in the stratum *h*. The probability of selecting the *i*^th^ EA in stratum *h* from the sampling frame is calculated as follows:

$$P_{1hi}=\frac{n_{h} M_{hi} }{\sum M_{hi} }$$

Let $s_{hi}$ be the proportion of households in the selected segment compared to the total number of households in EA *i* in stratum *h* if the EA is segmented, otherwise$s_{hi}=1$. Let $L_{hi}$ be the number of households listed in the household listing operation in cluster *i* in stratum *h*, let $m_{hi}$ be the number of households selected in the cluster. The second stage’s selection probability for each household in the cluster is calculated as follows:

$$P_{2hi}=\frac{m_{hi}}{L_{hi}}\times s_{hi}$$

The overall selection probability of each household in cluster *i* of stratum *h* is therefore the production of the selection probabilities:

$$P_{hi}=P_{1hi}\times P_{2hi}$$

Therefore, the design weight for each household in cluster *i* of stratum *h* is the inverse of its overall selection probability:

$$W_{hi}=1/P_{hi}$$

A spreadsheet containing all the sampling parameters and selection probabilities will be prepared to facilitate the calculation of the design weights. Design weights will be adjusted for household non-response and as well as for individual non-response to get the sampling weights, for women and men surveys respectively. The differences of the household sampling weights and the individual sampling weights are introduced by individual non-response.

The final sampling weights will be normalized in order to give the total number of un-weighted cases equal to the total number of weighted cases at national level, for both household weights and individual weights, respectively. The normalized weights are relative weights which are valid for estimating means, proportions and ratios, but not valid for estimating population totals and for pooled data. The sampling weights for the various indicators would be calculated in a similar way for adult female and male at the national level, in order that the prevalence calculated for adult female and male together are valid. Sampling errors will be calculated for selected indicators for the national sample, urban and rural areas separately, and for each of the district.

**Table S2.** NDVI Classification in Greater Accra Metropolitan Area for the years 2000, 2015 and 2020

| **NDVI Classes** | **NDVI Range** | **Pixel count** | **NDVI %** | **Area (km^2^)** | **Area %** |
| --- | --- | --- | --- | --- | --- |
|  | **2000** | | | | |
| Bare/No Vegetation | -1 - 0.1 | 14,735.00 | 30.4% | 10,657.84 | 26.1% |
| Unhealthy Vegetation | - 1. - 0.33 | 11,027.00 | 22.7% | 17,070.19 | 41.9% |
| Moderately Healthy Vegetation | 0.33 – 0.66 | 11,386.00 | 23.5% | 8,542.86 | 20.9% |
| Healthy Vegetation | 0.66 – 0.8 | 6,428.00 | 13.2% | 3,017.75 | 7.4% |
| Very Healthy Vegetation | 0.8 – 1.0 | 4,955.00 | 10.2% | 1,491.16 | 3.7% |
| Total |  | 48,531.00 | 100.0% | 40,779.81 | 1 |
|  | **2015** | | | | |
| Bare/No Vegetation | -1 - 0.1 | 44,992.00 | 25.1% | 2609.15 | 19.00% |
| Unhealthy Vegetation | - 0.33 | 46229 | 25.8% | 5307.95 | 38.66% |
| Moderately Healthy Vegetation | 0.33 – 0.66 | 37195 | 20.8% | 2961.47 | 21.57% |
| Healthy Vegetation | 0.66 – 0.8 | 29468 | 16.4% | 2179.12 | 15.87% |
| Very Healthy Vegetation | 0.8 – 1.0 | 21,287.00 | 11.9% | 671.35 | 4.89% |
| Total |  | 179,171.00 | 100.0% | 13,729.05 | 100.0% |
|  | **2020** | | | | |
| Bare/No Vegetation | -1 - 0.1 | 78,180.00 | 39.4% | 9163.30 | 47.71% |
| Unhealthy Vegetation | - 0.33 | 85438 | 43.1% | 9133.52 | 47.55% |
| Moderately Healthy Vegetation | 0.33 – 0.66 | 9897 | 5.0% | 396.70 | 2.07% |
| Healthy Vegetation | 0.66 – 0.8 | 11922 | 6.0% | 324.82 | 1.69% |
| Very Healthy Vegetation | 0.8 – 1.0 | 12,999.00 | 6.6% | 189.33 | 0.99% |
| Total |  | 198,436.00 | 100.0% | 19,207.67 | 100.00% |
