# Supplementary material for: Socio-demographic and neighbourhood factors influencing urban green space use and development at home: A population-based survey in Accra, Ghana
Source: PLoS One. 2023 Jun 23;18(6):e0286332. doi: 10.1371/journal.pone.0286332 (PMC10289371; doi:10.1371/journal.pone.0286332)
Supplement: Attachment — Submitted filename: Review Report.docx [file pone.0286332.s002.docx]

PLOS ONE

PONE-D-22-22097

Socio-demographic and neighbourhood factors influencing urban green space use and

development at home: A population-based survey in Accra, Ghana

The study combines individual level primary data with contextual secondary data to examine factors associated with access and use of green spaces in Accra, the capital city of Ghana. This is a timely and important study given its relevance to awareness of the fast depleting green spaces in urban environments. However, there are some critical issues that need addressing.

Abstract, provide a brief background of the study area (Accra) to help the reader appreciate the context of the study.

Introduction – Paragraph 2, the authors should use statistical data from the literature to evidence the decline in urban green spaces.

Line 73, “…, it often does NOT translate …” Not is missing.

Line 87 – 88, the authors claim that two studies have been done in Accra but they are narrow in scope. The authors should explain why those studies were narrow in scope, how their study will help fill those gaps.

Introduction, the authors need to provide a convincing justification for the choice of Accra for such a study. Note that the study could have been done in any of the cities in the country.

Lines 107 – 111, the authors should provide a scientific justification with literature evidence to support the classification of population density and income levels of districts. What does the researchers mean by high-income neighbourhoods and how does it translate into income levels?

Lines 116 – 127, when was the data collected?, what data was collected? What were the background characteristics, e.g., age of the respondent, etc…?

Lines 121-123, given the sampling allocation procedure described in Supplementary Table 1, it is not correct to state that “…10 households were selected from the listed households…”. Rather say, 2000 households were selected, allocated proportionately to the number of households in each district.

Lines 121- 123, Describe the sampling procedure used to select 10 households from each EA, and how that leads to a selection of a representative sample for generalisability.

Lines 123-126, What was the relevance of interviewing two people from the same household since it introduces dependence and violates the assumption of statistical independence? Note that interviewing household members separately does not rule out the problem of statistical dependence, since household members have similar or same exposure to access to green space and other factors.

Lines 123-126, What criteria was used to select the second person from the household?

Line 125, the study conducted is quantitative in nature, however, the authors claim that a semi-structured instrument was used for data collection. How is this possible since semi-structured interview guides are used for qualitative studies?

Line 126, define GAMA before using the abbreviation.

Lines 132 – 141, there is potential high multicollinearity in the indicators and scores described. Summing to generate an overall score, high scores in one indicator has the potential to cancel out the scores for another indicator, for example, scoring 2 + 2 + 2 + 2 = 8, also 3 + 3 + 1 + 1 = 8. In this case, using a method of differential weighting such as factor analysis circumvents this problem, and allocate weights due to variation (variances) in the responses. The authors should explain the robustness and limitations of the approach they adopted.

Lines 144 – 158, the authors should explain what NVDI is, what they were used to proxy in this study, with a justification and how the 2000, 2015 and 2020 data were linked to a study conducted in 2021/22?

Why were some of the contextual factors extracted at the district level and others at the community level?

Ethical consideration

The authors stated that verbal informed consent was obtained. They should indicate how it was documented and witnessed. If the study included minors, state how informed consent was obtained. State the date ethical clearance was obtained, the period of data collection, the clearance number to show proof that clearance was obtained before data collection.

Statistical analysis

- “Multivariable binary logistic regression adjusting for potential confounders…” This indicates that there were primary factors upon which the research focuses. Thus, clarify what the primary factors and confounders are.
- What is (are) the outcome variable(s) of interest? Clearly state and indicate how they were classified into 0 and 1 (binary).
- As indicated earlier, two respondents were sampled from each household. This introduces dependence in the data structure which results in a more complex error structure than in a standard ordinary least squares model. When a standard ordinary least squares model is fitted in such case, it could result in biased standard errors, thus affect the model outcome. Explain how nesting within households was accounted for in this analysis. Could adopt multilevel (mixed effects) modelling approaches.

Results

- Tables 3 should rather focus on the main outcome (dependent) variables used for the multivariable analyses to give clarity. Table 5 should focus on the independent variables, classified into primary factors and confounders. Tables 3, 4, 5,.. do not add much to achieving the objectives of the study but rather complicates the story, particularly with the varying and low sample sizes in some cases. Also, those indicators presented in Tables 3, 4 and 5 but are not used in the main (multivariable) analysis should also be excluded. They provide some detail but do not add to achieving the objectives of the paper.
- Some of the variables, e.g., NDVI, population density, etc… were employed as contextual factors but the level at which these were link to the individual data for analysis are not explained.
- Interpretation of odds ratios
  - the authors should be specific of what the odds are. For example, rounding 4.53 to 5 is not appropriate since the difference of the effects could be substantial.
  - Odds should be interpret as odds, for example, females have higher odds of 4.53 of using green space when compared to males.
  - The authors should explain the difference between odds ratios, adjusted odds ratios and crude odds ratios and use them consistently.
- Tables 6, 7 and 8 the authors should use stars (*) to indicate the level of significance to provide clarity on which categories significantly different from others.
- Was the analysis presented in Tables 7 and 8 conducted separately? If yes, then that is appropriate. The socio-demographic factors and neighbourhood factors do not influence green space development independently. For example, persons with higher education maybe more likely to reside in high-income and greener neighbourhoods thus influencing their development of green space. As indicated earlier, the neighbourhood (contextual) factors should be linked to the individual data and the analysis conducted and presented in one table.
- Also, account for the contextual factors in the analysis for use of green space.
- Why are the adjusted odds ratios for population density, income level of district and neighbourhood greenness not reported. Were they statistically insignificant? Please, clarify.
- Lines 388-428, the discussions are missed placed. You do not conduct the analysis before you discuss or describe the variables. You discuss the variables in the data section before you conduct the analysis using the variables.
- Validity issues – these should be discussed in the data section not discussion of the results.

Discussion – Synthesis with previous knowledge

Some sections of the discussion re-present the findings. The focus of the discussions should be on the synthesis and new knowledge.

Lines 545-548, the attribution made by the authors could not be correct. In the context of Ghana, persons of higher education may be engaged in activities that limiting the time to access green space but not that they are not aware of the benefits of green space as claimed by the authors. In this regard, it is important that the authors use literature evidence to attribute their findings

The authors do not use any literature evidence to support or dispute the discussion in the last three paragraphs but just present the results. It is strongly recommended that the discussions focuses on the main findings and literature evidence are used to support the findings. In this regard, the discussions could be shortened.
